# Comparison of Children versus Adults Undergoing Mini-Percutaneous Nephrolithotomy: Large-Scale Analysis of a Single Institution

**DOI:** 10.1371/journal.pone.0066850

**Published:** 2013-06-24

**Authors:** Guohua Zeng, Zhijian Zhao, ShawPong Wan, Wen Zhong, Wenqi Wu

**Affiliations:** 1 Department of Urology, Minimally Invasive Surgery Center, the First Affiliated Hospital of Guangzhou Medical University, Guangzhou, China; 2 Guangdong Provincial Key Laboratory of Urology, Guangzhou, China; The University of Manchester, United Kingdom

## Abstract

**Objective:**

As almost any version of percutaneous nephrolithotomy (PCNL) was safely and efficiently applied for adults as well as children without age being a limiting risk factor, the aim of the study was to compare the different characteristics as well as the efficacy, outcome, and safety of the pediatric and adult patients who had undergone mini-PCNL (MPCNL) in a single institution.

**Methods:**

We retrospective reviewed 331 renal units in children and 8537 renal units in adults that had undergone MPCNL for upper urinary tract stones between the years of 2000–2012. The safety, efficacy, and outcome were analyzed and compared.

**Results:**

The children had a smaller stone size (2.3 vs. 3.1 cm) but had smilar stone distribution (number and locations). The children required fewer percutaneous accesses, smaller nephrostomy tract, shorter operative time and less hemoglobin drop. The children also had higher initial stone free rate (SFR) (80.4% vs. 78.6%) after single session of MPCNL (p<0.05); but no difference was noted in the final SFR (94.7% vs. 93.5%) after auxiliary procedures. The complication rate (15.6% vs. 16.3%) and blood transfusion rate (3.1% vs. 2.9%) were similar in both group (p>0.05). Both groups had low rate of high grade Clavien complications. There was no grade III, IV, V complications and no angiographic embolization required in pediatric group. One important caveat, children who required multiple percutaneous nephrostomy tracts had significant higher transfusion rate than in adults (18.8% vs. 4.5%, p = 0.007).

**Conclusions:**

This contemporary largest-scale analysis confirms that the stone-free rate in pediatric patients is at least as good as in adults without an increase of complication rates. However, multiple percutaneous nephrostomy tracts should be practiced with caution in children.

## Introduction

Urolithiasis in children is an important health issue. The optimal management of for pediatric stone disease is still evolving. The treatment should not impair the growth and function of the young kidneys. Currently extracorporeal shock wave lithotripsy (ESWL) is often utilized as the first line treatment. However, the long term safety of ESWL has been questioned. Furthermore due to the higher incidence of metabolic and anatomical abnormalities in the pediatric stone patients, any residual stone after ESWL can more lead to recurrence [1]–[2]. The other main concerns in children are to minimize the need for retreatment, and thus any possible treatment options to achieve a stone-free status in this age group are very important and should not be limited or precluded. The ideal treatment should therefore be minimally invasive with high stone free rate (SFR) and lower retreatment rate [1].

Minimally invasive percutaneous nephrolithotomy (MPCNL), a modified standard PCNL with smaller percutaneous tract size (14–20F), has been proven to be safe and efficacious in treating both pediatric and adult patients [3]–[5]. However, due to the low number of pediatric patients requiring MPCNL and the higher technical demand for such procedure, MPCNL is still not commonly performed outside of tertiary hospitals. In children, the pelvicalyceal system is less robust and the tolerance for blood loss is more limited; thus there is much less a margin for error, an important challenge to the endourologist.

Previous reports of pediatric PCNL tended to use the different surgical equipment in different age groups to categorize the results [6]–[9]. In current study, our goal was to compare the different characteristics as well as the outcome of the pediatric and adult patients who had undergone MPCNL in a single institution using the same surgical equipment and technique.

## Patients and Methods

This study was approved by the Ethics Review Board of Guangzhou Medical College, Guangzhou, China. In addition written informed consent was obtained from each patient and/or the guardians, especially in cases of the child participants. We retrospectively reviewed the data of 8868 renal units in 7420 patients with upper urinary tract calculi who underwent one-stage MPCNL. MPCNL’s were performed in accordance to our own guidelines and techniques. Indications for MPCNL were mainly based on the stone sizes, their location, and the presence or absence of infection and hydronephrosis. Urine culture, serum biochemistry, and coagulation tests were obtained in every patient. Radiological studies performed included intravenous urography (IVU) and urinary tract ultrasound, and in selected cases, non contrast computed tomography (CT). Broad spectrum prophylactic antibiotics were administered one hour preoperatively to patients with sterile urine. Patients with culture proven bacteriuria were first treated with antibiotics according to the antibiogram.

All MPCNL were performed using standardized single session technique. The procedures were performed either under general or continuous epidural anesthesia. The patients were first placed in lithotomy position. A 5Fr or 6Fr ureteral catheter is inserted in a retrograde fashion. The patients were then turned into prone position for the MPCNL. Percutaneous access was obtained using either C-arm fluoroscopy or ultrasonography by the surgeon. After renal puncture, a flexible tip guidewire was next inserted. The nephrostomy tract was established using fascial dilators up to 14 - 20 Fr. according to the clinical need. This was followed by placement of a corresponding size peelable sheath. For complex stone where multiple percutaneous tracts were deemed necessary; these accesses were created during the same session. 8/9.5 Fr. semirigid ureteroscope (Richard Wolf, Germany) was the endoscope of choice. Holmium:YAG laser and/or pneumatic lithotripters were used for stone fragmentation. Pulse perfusion pump was used for irrigation and for flushing out small (<3 mm) stone fragments. Stone forceps was used for removal of larger fragments. At the conclusion of the procedure, a nephrostomy tube and a 4 to 6 Fr, double pigtail ureteral stent were placed. Patients with bilateral upper urinary tract stones were treated in consecutive session with minimum 4–5 days interval. No simultaneous bilateral MPCNL was performed.

Routine KUB, if necessary, noncontrast CT was obtained 24–48 hours postoperatively in every patient. The nephrostomy tubes were remove in patients who were either stone free or with clinically insignificant stone (</ = 4 mm), and also were afebrile, asymptomatic, and absence of significant drainage from the nephrostomy tube. Patients were generally discharged home after removal of the nephrostomy tubes as per our local custom and practice. The double pigtail ureteral stents were removed 2–4 weeks later in outpatient clinic. A second-look MPCNL session, if indicated, was performed when the initial procedure was deemed less than satisfactory due to significant residual stones discovered postoperatively. ESWL, ureteroscopy (URS), and occasional repeat MPCNL were considered as auxiliary procedure alternatives when indicated.

Patients were divided into pediatric, 0–14 years old, and adult, >14 years old, groups in accordance to World Health Organization (WHO) age classification criteria for this comparison study.

Preoperative data collected included previous stone intervention history, co-morbidities, stone size, and stones distribution (number and locations). Stone size was assessed as the largest diameter on the radiological study. Intraoperative data included operation time, renal puncture location, size and number of the nephrostomy tract, and any intraoperative complications. Operation time was defined as the time between the first renal puncture to the completion of the stone removal. Postoperative parameters obtained included SFR, any postoperative complications, hemoglobin drop, blood transfusion, and any re-treatment. Initial SFR was defined as either stone free or with asymptomatic and clinically insignificant residual stone of </ = 4 mm on KUB or CT at 24–48 hour post-MPCNL. Final SFR was defined same as initial SFR but at one month post operatively and after any repeat MPCNL or auxiliary procedures. Complications were recorded according to modified Clavien classification system [10].

Comparisons for proportions between groups were performed with chi-square or Fisher’s exact probability test. Comparisons for continuous variables were performed with Student’s t test or Mann–Whitney U test. P<0.05 was considered statistically significant.

## Results

7420 patients entered into this study. 331 (4.5%) were children and 7089 (95.5%) were adults. No bilateral upper tract stones requiring MPCNL in children. 1448 (20.4%) of the adult patients required bilateral procedures. A male dominance was seen in adults group, 58.1%, p = 0.036. The mean stone size was 2.3+/−0.6 cm in children and 3.1+/−0.9 cm in adults, p<0.001. The stone distribution (numbers and locations) were similar in both children and adults groups (table 1). As expected, children had significant lower co-morbidities, 0% vs. 24.6%. 109 (32.9%) of the children and 3048 (35.7%) of the adults had history of previous surgical intervention in the same kidney prior to coming to our institution. 6 vs. 292 had previous open surgery, 76 vs. 1445 had ESWL, 19 vs. 1122 had PCNL, 8 vs. 189 had URS, in children vs. adults respectively. Preoperative urine cultures were positive in 46 (13.9%) of the children and 1497 (17.5%) of the adults, p = 0.087. All the infections were treated with culture specific antibiotics. Demographic and preoperative characteristics of the patients were summarized in Table 1.

**Table 1:** Demographic and clinical preoperative characteristics.

Characteristics	Overall	Children	Adults	P
Number of patients	7420	331(4.5%)	7089(95.5%)	–
Number of renal units	8868	331(3.7%)	8537(96.3%)	–
Mean±SD age(range)(yr)	47.6±15.3(1–93)	7.8±3.9(0.7–14)	49.1±13.3(15–93)	<0.001
Number of sex:				0.036
Male	4291(57.8%)	173(52.3%)	4118(58.1%)	
Female	3129(42.2%)	158(47.7%)	2971(41.9%)	
Previous stone-related surgery:	3157(35.6%)	109 (32.9%)	3048(35.7%)	0.301
PCNL	1141(12.9%)	19(5.7%)	1122(13.2%)	
SWL	1521(17.2%)	76(23.0%)	1445(16.9%)	
Pyelolithotomy	298(3.4%)	6(1.8%)	292(3.4%)	
URS	197(2.1%)	8(2.4%)	189(2.2%)	
Comorbidities	2100(23.7%)	0	2100 (24.6%)	<0.001
Mean ±SD stone size(range) (cm)	3.1±0.9(1.2-7.4)	2.3±0.6(1.3-3.6)	3.1±0.9(1.2-7.4)	<0.001
Number of stones				0.382
Staghorn	2800(31.6%)	94(28.4%)	2706(31.7%)	
Multiple	3460(39.0%)	131(39.6%)	3329(39.0%)	
Single	2608(29.4%)	106(32.1%)	2502(29.3%)	
Stone locations:				0.334
Pelvis	1794(20.2%)	78 (23.7%)	1716(20.1%)	
Pelvis+calyx	5104(57.6%)	178(53.8%)	4926(57.7%)	
Calyx only	1671(18.8%)	66 (19.8%)	1605(18.8%)	
Upper ureter	299(3.4%)	9(2.7%)	290(3.4%)	
Positive preoperative urine culture	1543(17.4%)	46(13.9%)	1497(17.5%)	0.087

Operative characteristics were listed in Table 2. In summary: 315 (95.2%) children had single nephrostomy tract established for the MPCNL and 16 (4.8%) required two tracts. Of the total 347 nephrostomy tracts, 221 (63.7%) were created through the 11th intercostal space to access the calices. The remaining 126 (36.3%) were through a sub-12th rib approach. For adults, a total of 10801 nephrostomy tracts were established for 8537 cases of MPCNL. Single tract was carried out in 6627 (77.6%) cases, and 2 tracts in 1627(19.1%), 3 tracts in 231(2.7%), 4 tracts in 38(0.4%), 5 tracts in 15(0.2%). Similar to the children, majority, 7097 (65.7%), of the nephrostomy tracts were created throught the 11th intercostal space. The mean nephrostomy tract size was 17.1 Fr. for children and 18.5 Fr. for adults, p<0.001. The most common tract size for children was 16fr. and 18Fr. for adults, 43.8% and 54.1% respectively.

**Table 2:** Operative characteristics.

Characteristics	Overall	Children	Adults	P
N	8868	331	8537	
Number of tracts:				
Single	6942(78.3%)	315(95.2%)	6627(77.6%)	<0.001
Multiple:	1926(21.7%)	16(4.8%)	1910(22.4%)	
Two	1643(18.5%)	16(4.8%)	1627(19.1%)	
Three	231(2.6%)	0(0%)	231(2.7%)	
Four	38(0.4%)	0(0%)	38(0.4%)	
Five	15(0.2%)	0(0%)	15(0.2%)	
Total Number tracts:	11148	347	10801	
Puncture site:				0.436
Supracostal	7318(65.6%)	221(63.7%)	7097(65.7%)	
Infracostal	3830(34.4%)	126(36.3%)	3704(34.3%)	
Mean ±SD nephrostomy tract size(F)	18.49±1.43	17.05±1.54	18.54±1.41	<0.001
14F	413(3.7%)	24(6.9%)	389(3.6%)	
16F	594(5.3%)	152(43.8%)	442(4.1%)	
18F	5980(53.7%)	136(39.2%)	5844(54.1%)	
20F	4161(37.3%)	35(10.1%)	4126(38.2%)	
Stone fragmentation methods:				0.196
Pneumatic	8077(91.1%)	295(89.1%)	7812(91.2%)	
Laser	791(8.9%)	36(10.9%)	755(8.8%)	
Mean±SD operative time (range) (min)	84.2±28.4(26-215)	73.6±20.2(26-133)	84.6±28.6(30-215)	<0.001
Stone free rate:				
Initial stone-free rate	6436(72.6%)	266(80.4%)	6170(78.6%)	0.001
Final stone-free rate	8295(93.5%)	313(94.7%)	7982(93.5%)	0.44
Staged MPCNL/Auxiliary procedures				
None	7312(82.5%)	277(83.7%)	7035(82.4%)	0.548
URS	190(2.1%)	11(3.3%)	179(2.1%)	
PCNL	807(9.1%)	13(3.9%)	794(9.3%)	
ESWL	488(5.5%)	27(8.2%)	461(5.4%)	
Combined methods	71(0.8%)	3(0.9%)	68(0.8%)	
Mean ±SD hemoglobin drop (range) (gm/dl)	1.5±0.6(0.2-6.8)	1.4±0.73(0.2-3.5)	1.5±0.6(0.7-6.8)	0.043
Mean ± SD postoperative hospital stay (range) (d)	5.4±2.1 d (1-16d)	5.2±2.4d (1-13)	5.4±2.1 d (1-16d)	0.09
Overall complications rate	1443(16.3%)	51(15.6%)	1392(16.3%)	0.664

The mean operative time was significantly shorter in children than in adults, 73.6+/−20.2 (26-133) vs. 84.6+/−28.6 (30-215) minutes, p<0.001. The mean hemoglobin decrease was 1.4+/−0.73 (0.2-3.5) g/dl for children and 1.5+/−0.6 (0.7-6.8) g/dl for adults, p = 0.043.

SFR after single session of MPCNL (initial SFR) was 80.4% in children and 78.6% in adult, p = 0.001. The SFR increased to 94.7% in children and 93.5% in adults after auxiliary procedure (final SFR), p = 0.44. The postoperative hospital stay was similar in both groups, 5.2+/−2.4 days and 5.4+/−2.1 days, p = 0.09.

There were 1855 (20.9%) perioperative complications encountered in all the patients. We assessed the complications followed the Clavien classification, table 3. Overall, grade I was documented in 11.3%, grade II in 7.1%, grade III in 2.7%, and grade IV in 0.05%. There were 2 deaths in the adults group, grade V complication, for a mortality rate of 0.02%. The causes of death was disseminated intravascular coagulopathy (DIC) resulted from uncontrolled sepsis in one and myocardial infarction in the other. There was no difference in perioperative complications between the children and adults (p>0.05). There were however no grade IV or V complications in children. The blood transfusion rate was similar for children and adults, 3.1% for the former and 2.9% for the latter, p = 0.0902. Of interest, when we analyzed children who required multiple nephrostomy tracts; we did notice a significant increase in hemoglobin drop and transfusion rate as compared to the adults. The hemoglobin drop was 2.7+/−0.55 g/dl vs. 2.0+/−0.88 g/dl (p = 0.001) and transfusion rate was 18.8% vs. 4.5% (p = 0.007).

**Table 3:** Complications by modified*C*lavien classification.

Characteristics	Overall	Children	Adults	P
No. renal units	8868	331	8537	–
No. kidneys of complications	1443(16.3%)	51(15.6%)	1392(16.3%)	0.664
No. total complications*	1855(20.9%)	84(25.5%)	1771(20.7%)	–
% Grade I:	1004(11.4%)	56(16.9%)	948(11.1%)	
Post-operative pain	511(5.8%)	24(7.2%)	487(5.7%)	0.236
Vomit	111(1.3%)	9(2.7%)	102(1.2%)	0.014
Fever with antipyretic therapy	382(4.3%)	23(6.9%)	359(4.2%)	0.016
% Grade II:	630(7.1%)	23(6.9%)	607(7.1%)	
Infection needed antibiotic	372(4.2%)	13(3.9%)	359(4.2%)	0.85
Blood transfusion	258(2.9%)	10 (3.1%)	248(2.9%)	0.902
% Grade III:	241(2.7%)	5(1.5%)	236(2.8%)	
Bleeding requiring multiple bladder washout/irrigation	70(0.8%)	2(0.6%)	68(0.8%)	0.698
Embolization	4949(0.56%)	0	49(0.57%)	0.167
Sepsis requiring surgical intervention	1212(0.14%)	0	12(0.14%)	0.495
Pleural injury requiring chest tube	79(0.9%)	2 (0.6%)	77 (0.9%)	0.572
Colonic perforation	3 (0.035%)	0	3 (0.035%)	>0.999
Renal pelvic perforation	11(0.12%)	0	11(0.13%)	0.513
Perirenal abscess by pecutaneous drainage	17(0.2%)	1(0.3%)	16(0.18%)	0.64
% Grade IV	5(0.05%)	0	5(0.05%)	
Nephrectomy	2(0.02%)	0	2(0.02%)	>0.999
Sepsis causing organ injury	3(0.03%)	0	3(0.03%)	>0.999
% Grade V	2(0.02%)	0	2(0.02%)	
Death	2(0.02%)	0	2(0.02%)	>0.999

* Some cases experienced one or more complications.

## Discussion

The management of urolithiasis in children is still evolving. Any form of treatment necessitates balancing between the SFR and the morbidities associated with the procedure. ESWL has been a treatment choice for upper urinary tract stones of less than 20 mm in size. In children sometime stones greater 20 mm were also managed in this fashion. However, only 37-52% of children treated with ESWL were stone free at discharge. The percentage increased to 57-97% in 3 months after retreatment. The retreatment rate was 10.7-53.9% [1]. Afshar et al. reported that on a mean follow up of 48 months, 69% of children with residual stone fragments after ESWL would experience symptoms as well as an increase in stone size [2]. Of further concern, the potential long term side effects of ESWL therapy have been reviewed and debated. There were data seemed to suggest a possible increase in the risks of hypertension, diabetes mellitus, arteriole sclerosis, and long term renal tubular injury in patients treated ESWL [1]. Therefore, ESWL should be employed judiciously, especially in pediatric population.

PCNL is an effective treatment option for large upper urinary tract stones in children. However, the less robust and smaller pelvicalyceal system, the hypermobility of the kidney, and the lower tolerance of blood loss in children were some of the factors making this procedure more challenging. Recently a Clinical Research Office of the Endourological Society (CROES) Study showed children only constituted 3.8% of all PCNL performed. Only 25% of major tertiary centers reported doing pediatric PCNL [6]. Thus, this procedure may not have reached its full potential.

Many studies have shown the efficacy and safety of pediatric PCNL. We have also previously reported our experience using our version of MPCNL in infants as young as 7 months with complex stone disease [11]. There were many other literatures addressing pediatric PCNL. The SFR after PCNL in pediatric patients ranged from 59 to 98%. Samad et al. reported a 59% SFR after PCNL monotherapy. The mean stone burden was 3.1 cm. The SFR rate increased to 93.8% after adjunctive ESWL. Their transfusion rate was 3.6%. Bilen et al. reported a SFR of 90% in 188 consecutive PCNL [12]. In a series of 60 children (mean age 6 years old) underwent PCNL, Badawy et al. reported an 83.3% SFR after single treatment session [13]. In another series of 135 children, average age of 8.9 years old, Salah et al. reported a 98.5% of SFR. He also had minimum complications, 8% urine leakage and 0.7% transfusion [3]. PCNL can also be used effectively in the complex or staghorn stones in children. The SFR had been reported to be 73.6%, 89%, and 89.8% after single session in three separate studies. The SFR were further improved to 86.8%, 94.7% and 96% respectively after adjunctive ESWL [14]-[16]. Bilen et al. was also the first one to report using tubeless PCNL in children and they had a SFR of 91.6% with this technique [9].

Complication is the most important issue in any PCNL. In pediatric patients, the blood loss requiring transfusion was reported to be in 0.4-24% range. It correlated with stone burden, nephrostomy tract size, number of tracts, and operative time [4], [12], [14], [16]-[19]. Zeren used 18-30 Fr. nephrostomy tracts in pediatric PCNL. He reported a 87% SFR, a 30% postoperative fever, and a 24% transfusion rate [4]. Biden et al. demonstrated a higher transfusion in children with nephrostomy tracts of greater than 20 Fr.; whereas there no transfusion required with 14 Fr. Tract [12]. Guven et al. used single nephrostomy tract only PCNL in infants with complex renal stones. They noted higher hemoglobin drop when the tract size was greater than 20Fr [17]. One should keep in mind that a 24Fr. tract in infant is equivalent to about 72 Fr. in adults [18]. Therefore, the optimal size for nephrostomy tract in children probably should be between 14 to20 Fr. Ozden [14], Desai [16], and Manohar [19] reported average decrease of hemoglobin of 1.6g/dL, 1.9 g/dL, and 2.2 g/dL respectively in pediatric PCNL for complex calyceal and staghorn calculi. The authors noted that the number and the sizes of the nephrostomy tracts were significantly correlated to the hemoglobin decrease; each of the p value was <0.001. In our series, we also noted a statistically significant increase in blood loss and transfusion rate in patients with multiple nephrostomy tracts. The local damage to the renal cortex and parenchyma during PCNL should also be considered. Samad et al. reported cortical defects (renal scars) on Tc-DMSA (Tc99) scan in 10 (17%) of the 60 renal units [20]. Last but not the least, the radiation exposure to the pediatric patient population should be minimized. Obviously the less the renal puncture, the less the radiation is required.

We prefer an 11th or higher intercostal space for establishing the nephrostomy tract in children with complex calyceal or staghorn stones. The puncture is usually aimed at middle calyces. We found this approach provide good accesses to the upper ureter, the renal pelvis, as well as to the most of the calyces. The safety and efficacy of this approach was also confirmed by El-Nahas [8] and Anand et al [21].

We have recorded shorter operative time in the children. This may be explained by Falahatkar et al study. The authors concluded that number of nephrostomy tracts, stone burden, and location of calyx for access (upper>lower>middle) were all positively factors influenced the operative time [22].

The cultural difference in the Chinese health care kept our postoperative hospital stay longer than many other countries. In the U.S. as well as many other western nations, the removal of nephrostomy tube and management of nephrostomy tract drainage were done on outpatient settings; whereas, in China, it is socially unacceptable for the patients to go home with tube in place or with urine drainage. On the other hand, our hospital stay was only a fraction of cost as compared to the others.

Our current study is the first to use modified Clavien system to compare the complications of MPCNL in children vs. adults. Complications according to Clavien classification were reported as 29.2% in adults by Tefekli et al [10]and 30% by Ozden et al in children [23]. Our overall renal complication rates were 15.6% for children and 16.3% for adults, p = 0.664. The grade I complication (vomiting and transient fever) rate was higher in children. This however could be attributed to anesthesia. High grade complications were low in both groups. There was no grade IV or V complication, and no selective angiographic embolization required in the children.

In conclusion, we reaffirmed that MPCNL is a safe and effective treatment modality for children with large upper urinary tract stone disease. There was no significant difference in the surgical outcome and complications as compared to adult population. However, there were also some notable distinctions. Pediatric patients had lower co-morbidities but same urinary tract infection rate. Children had smaller stone burden but with similar distribution as adults. Of interest, both groups had similar prior surgical intervention rate. Pediatric patients required fewer and smaller nephrostomy tracts as well as less operative time. There was no Clavien grade IV or V complications in children and none required angiographic embolization. There was higher initial SFR in children; but this difference became insignificant after adjunctive procedures. Children required multiple nephrostomy tracts did experience more blood loss (p<0.005) and probably more transfusion (p = 0.007). Therefore, multiple tracts should be avoided as much as possible. We found most pediatric stones could be managed with single nephrostomy tract. In our experience, an 11th intercostal space puncture through a middle calyx was a superb approach. The heterogenous nature of our patient population might have an impact on the outcome of our study. However, despite this limitation, our large scale study reflected the real life clinical situations. We believe this report did demonstrate that MPCNL could be employed as a first line treatment for large upper urinary tract stones in Children.
